# 2-Chloro-*N*-methyl-*N*-phenyl­acetamide

**DOI:** 10.1107/S1600536810050427

**Published:** 2010-12-11

**Authors:** Li-Hua Zhi, Wei-Na Wu, Xiao-Xia Li, Yan-Wei Li, Yuan Wang

**Affiliations:** a Department of Physics and Chemistry, Henan Polytechnic University, Jiaozuo 454000, People’s Republic of China; b Institute of Functional Materials, Jiangxi University of Finance & Economics, Nanchang 330013, People’s Republic of China

## Abstract

In the title compound, C_9_H_10_ClNO, the non-H atoms, excluding the phenyl group, are almost coplanar (r.m.s. deviation of the non-H atoms = 0.1015 Å). The dihedral angle formed between this plane and the benzene ring is 87.07 (5)°. Weak inter­molecular C—H⋯O inter­actions help to stabilize the packing.

## Related literature

For the synthesis of lanthanide complexes with amide-type ligands, see: Wu *et al.* (2008 ▶). For related a structure, see: Yuan *et al.* (2010 ▶).
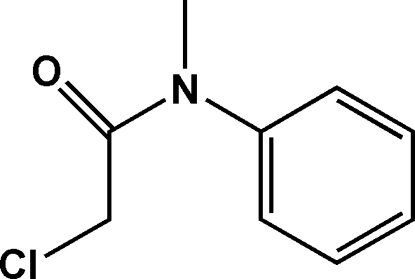


## Experimental

### 

#### Crystal data

C_9_H_10_ClNO
*M* *_r_* = 183.63
Monoclinic, 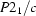

*a* = 7.3391 (12) Å
*b* = 6.5898 (10) Å
*c* = 18.941 (3) Å
β = 91.192 (9)°
*V* = 915.9 (2) Å^3^
*Z* = 4
Mo *K*α radiation
μ = 0.37 mm^−1^
*T* = 296 K
0.26 × 0.21 × 0.18 mm

#### Data collection

Bruker SMART CCD diffractometer
Absorption correction: multi-scan (*SADABS*; Bruker, 2007 ▶) *T*_min_ = 0.912, *T*_max_ = 0.936
9758 measured reflections
3003 independent reflections
1869 reflections with *I* > 2σ(*I*)
*R*_int_ = 0.021

#### Refinement

*R*[*F* ^2^ > 2σ(*F* ^2^)] = 0.044
*wR*(*F* ^2^) = 0.136
*S* = 1.04
3003 reflections
110 parameters
H-atom parameters constrained
Δρ_max_ = 0.24 e Å^−3^
Δρ_min_ = −0.30 e Å^−3^

### 

Data collection: *APEX2* (Bruker, 2007 ▶); cell refinement: *SAINT* (Bruker, 2007 ▶); data reduction: *SAINT*; program(s) used to solve structure: *SHELXS97* (Sheldrick, 2008 ▶); program(s) used to refine structure: *SHELXL97* (Sheldrick, 2008 ▶); molecular graphics: *SHELXTL* (Sheldrick, 2008 ▶); software used to prepare material for publication: *SHELXTL*.

## Supplementary Material

Crystal structure: contains datablocks I, global. DOI: 10.1107/S1600536810050427/vm2064sup1.cif

Structure factors: contains datablocks I. DOI: 10.1107/S1600536810050427/vm2064Isup2.hkl

Additional supplementary materials:  crystallographic information; 3D view; checkCIF report

## Figures and Tables

**Table 1:** Hydrogen-bond geometry (Å, °)

*D*—H⋯*A*	*D*—H	H⋯*A*	*D*⋯*A*	*D*—H⋯*A*
C2—H2⋯O1^i^	0.93	2.58	3.4356 (19)	154

Symmetry code: (i) 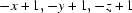.

**Fig. 1.**
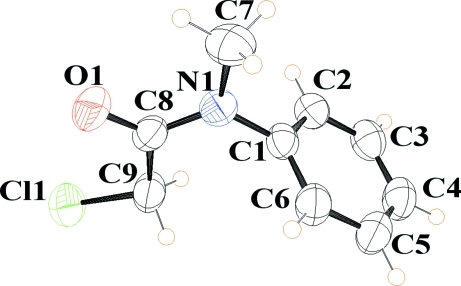
The molecular structure shown with 50% probability displacement ellipsoids.

## supplementary crystallographic information

### Comment

The luminescent properties of lanthanide complexes with amide type ligands have been investigated in our previous work (Wu *et al.*, 2008). As part of our ongoing studies of the amide type ligands, the title compound was synthesized and characterized by X-ray diffraction.

In the title compound (Fig. 1), the C—N bond lengths are shorter than those observed in a similar compound (Yuan *et al.*,2010). The non-hydrogen atoms excluding the phenyl group are almost coplanar (r.m.s. deviation of the non-hydrogen atoms being 0.1015 Å). The dihedral angle formed between this plane and the benzene ring (r.m.s. deviation 0.0021 Å) is 87.07 (5)°.

As expected, there are no classic hydrogen bonds in the structure. However, there is a weak intermolecular C2—H2···O1 hydrogen bond stabilizing the packing. An intramolecular C7—H7A···O1 hydrogen bond is also present (Table 1).

### Experimental

A chloroform solution containing chloroacetyl chloride (2.26 g, 0.02 mol) was added dropwise to a solution of *N*-methylbenzenamine (2.14 g, 0.02 mol) and pyridine (2.60 g, 0.03 mol) in chloroform (20 ml) under stirring on an ice-water bath. The reaction mixture was stirred at room temperature for 3.5 h. A solid product was separated from the solution by suction filtration, purified by succesive washing with water, 0.5 mol/*L* HCl, 0.5 mol/*L* NaOH and distilled water, respectively. Colourless block crystals were obtained by slow evaporation of the ethanol solution at room temperature.

### Refinement

The H atoms were placed at calculated positions and refined in riding mode, with the carrier atom-H distances = 0.93 Å for aryl, 0.97 for methylene, 0.96 Å for the methyl. The *U*_iso_ values were constrained to be 1.5*U*_eq_ of the carrier atom for the methyl H atoms and 1.2*U*_eq_ for the remaining H atoms.

### Crystal data

**Table:**

C_9_H_10_ClNO	*F*(000) = 384
*M**_r_* = 183.63	*D*_x_ = 1.332 Mg m^−^^3^
Monoclinic, *P*2_1_/*c*	Mo *K*α radiation, λ = 0.71073 Å
Hall symbol: -P 2ybc	Cell parameters from 3217 reflections
*a* = 7.3391 (12) Å	θ = 2.8–25.4°
*b* = 6.5898 (10) Å	µ = 0.37 mm^−^^1^
*c* = 18.941 (3) Å	*T* = 296 K
β = 91.192 (9)°	Block, colourless
*V* = 915.9 (2) Å^3^	0.26 × 0.21 × 0.18 mm
*Z* = 4	

### Data collection

**Table:**

Bruker SMART CCD diffractometer	3003 independent reflections
Radiation source: fine-focus sealed tube	1869 reflections with *I* > 2σ(*I*)
graphite	*R*_int_ = 0.021
φ and ω scans	θ_max_ = 31.5°, θ_min_ = 2.2°
Absorption correction: multi-scan (*SADABS*; Bruker, 2007)	*h* = −10→9
*T*_min_ = 0.912, *T*_max_ = 0.936	*k* = −9→9
9758 measured reflections	*l* = −26→27

### Refinement

**Table:**

Refinement on *F*^2^	Primary atom site location: structure-invariant direct methods
Least-squares matrix: full	Secondary atom site location: difference Fourier map
*R*[*F*^2^ > 2σ(*F*^2^)] = 0.044	Hydrogen site location: inferred from neighbouring sites
*wR*(*F*^2^) = 0.136	H-atom parameters constrained
*S* = 1.04	*w* = 1/[σ^2^(*F*_o_^2^) + (0.0656*P*)^2^ + 0.0768*P*] where *P* = (*F*_o_^2^ + 2*F*_c_^2^)/3
3003 reflections	(Δ/σ)_max_ < 0.001
110 parameters	Δρ_max_ = 0.24 e Å^−^^3^
0 restraints	Δρ_min_ = −0.30 e Å^−^^3^

### Special details

**Table:**

Geometry. All e.s.d.'s (except the e.s.d. in the dihedral angle between two l.s. planes) are estimated using the full covariance matrix. The cell e.s.d.'s are taken into account individually in the estimation of e.s.d.'s in distances, angles and torsion angles; correlations between e.s.d.'s in cell parameters are only used when they are defined by crystal symmetry. An approximate (isotropic) treatment of cell e.s.d.'s is used for estimating e.s.d.'s involving l.s. planes.
Refinement. Refinement of *F*^2^ against ALL reflections. The weighted *R*-factor *wR* and goodness of fit *S* are based on *F*^2^, conventional *R*-factors *R* are based on *F*, with *F* set to zero for negative *F*^2^. The threshold expression of *F*^2^ > σ(*F*^2^) is used only for calculating *R*-factors(gt) *etc*. and is not relevant to the choice of reflections for refinement. *R*-factors based on *F*^2^ are statistically about twice as large as those based on *F*, and *R*- factors based on ALL data will be even larger.

### Fractional atomic coordinates and isotropic or equivalent isotropic displacement parameters (Å^2^)

**Table:**

	*x*	*y*	*z*	*U*_iso_*/*U*_eq_	
Cl1	0.13525 (7)	0.14663 (8)	0.40483 (2)	0.07202 (19)	
N1	0.29635 (16)	0.47779 (19)	0.56727 (6)	0.0477 (3)	
C1	0.2909 (2)	0.3393 (2)	0.62581 (7)	0.0435 (3)	
C8	0.22967 (19)	0.4317 (2)	0.50259 (7)	0.0457 (3)	
C2	0.4463 (2)	0.2337 (2)	0.64621 (7)	0.0494 (3)	
H2	0.5533	0.2483	0.6212	0.059*	
C6	0.1317 (2)	0.3191 (3)	0.66275 (8)	0.0542 (4)	
H6	0.0279	0.3909	0.6489	0.065*	
O1	0.21932 (15)	0.55459 (18)	0.45449 (6)	0.0632 (3)	
C4	0.2821 (2)	0.0864 (3)	0.74123 (8)	0.0605 (4)	
H4	0.2794	0.0017	0.7805	0.073*	
C9	0.1674 (2)	0.2147 (3)	0.49372 (7)	0.0566 (4)	
H9A	0.0539	0.1962	0.5182	0.068*	
H9B	0.2575	0.1252	0.5154	0.068*	
C3	0.4407 (2)	0.1065 (3)	0.70395 (8)	0.0574 (4)	
H3	0.5442	0.0341	0.7178	0.069*	
C5	0.1284 (2)	0.1911 (3)	0.72052 (8)	0.0620 (4)	
H5	0.0214	0.1758	0.7455	0.074*	
C7	0.3571 (3)	0.6832 (3)	0.58295 (10)	0.0643 (4)	
H7A	0.3770	0.7546	0.5396	0.096*	
H7B	0.4686	0.6784	0.6103	0.096*	
H7C	0.2656	0.7523	0.6093	0.096*	

### Atomic displacement parameters (Å^2^)

**Table:**

	*U*^11^	*U*^22^	*U*^33^	*U*^12^	*U*^13^	*U*^23^
Cl1	0.0829 (3)	0.0883 (4)	0.0447 (2)	−0.0124 (2)	−0.0002 (2)	−0.00826 (18)
N1	0.0533 (7)	0.0396 (7)	0.0501 (6)	−0.0039 (5)	−0.0009 (5)	0.0047 (5)
C1	0.0526 (8)	0.0397 (7)	0.0379 (6)	−0.0028 (6)	−0.0028 (5)	−0.0021 (5)
C8	0.0450 (7)	0.0475 (8)	0.0446 (7)	0.0003 (6)	0.0043 (5)	0.0089 (5)
C2	0.0499 (8)	0.0490 (9)	0.0494 (7)	−0.0004 (7)	−0.0012 (6)	−0.0011 (6)
C6	0.0542 (9)	0.0634 (10)	0.0449 (7)	0.0054 (7)	0.0002 (6)	0.0026 (6)
O1	0.0722 (7)	0.0610 (7)	0.0563 (6)	−0.0022 (6)	−0.0001 (5)	0.0232 (5)
C4	0.0796 (11)	0.0583 (10)	0.0436 (8)	−0.0018 (8)	−0.0030 (7)	0.0096 (6)
C9	0.0768 (10)	0.0539 (9)	0.0389 (7)	−0.0095 (8)	−0.0006 (7)	0.0029 (6)
C3	0.0646 (10)	0.0537 (9)	0.0533 (8)	0.0065 (8)	−0.0121 (7)	0.0034 (7)
C5	0.0650 (10)	0.0755 (12)	0.0459 (8)	−0.0011 (9)	0.0085 (7)	0.0070 (7)
C7	0.0686 (11)	0.0438 (9)	0.0803 (11)	−0.0076 (7)	−0.0038 (9)	0.0011 (8)

### Geometric parameters (Å, °)

**Table:**

Cl1—C9	1.7537 (15)	C6—H6	0.9300
N1—C8	1.3446 (18)	C4—C5	1.373 (3)
N1—C1	1.4372 (17)	C4—C3	1.381 (2)
N1—C7	1.454 (2)	C4—H4	0.9300
C1—C6	1.380 (2)	C9—H9A	0.9700
C1—C2	1.384 (2)	C9—H9B	0.9700
C8—O1	1.2203 (16)	C3—H3	0.9300
C8—C9	1.509 (2)	C5—H5	0.9300
C2—C3	1.379 (2)	C7—H7A	0.9600
C2—H2	0.9300	C7—H7B	0.9600
C6—C5	1.382 (2)	C7—H7C	0.9600
			
C8—N1—C1	122.95 (12)	C8—C9—Cl1	112.56 (10)
C8—N1—C7	120.05 (13)	C8—C9—H9A	109.1
C1—N1—C7	116.56 (12)	Cl1—C9—H9A	109.1
C6—C1—C2	120.72 (13)	C8—C9—H9B	109.1
C6—C1—N1	119.34 (13)	Cl1—C9—H9B	109.1
C2—C1—N1	119.90 (13)	H9A—C9—H9B	107.8
O1—C8—N1	123.09 (14)	C2—C3—C4	120.20 (15)
O1—C8—C9	122.12 (13)	C2—C3—H3	119.9
N1—C8—C9	114.78 (12)	C4—C3—H3	119.9
C3—C2—C1	119.30 (14)	C4—C5—C6	120.32 (15)
C3—C2—H2	120.4	C4—C5—H5	119.8
C1—C2—H2	120.4	C6—C5—H5	119.8
C1—C6—C5	119.29 (15)	N1—C7—H7A	109.5
C1—C6—H6	120.4	N1—C7—H7B	109.5
C5—C6—H6	120.4	H7A—C7—H7B	109.5
C5—C4—C3	120.16 (15)	N1—C7—H7C	109.5
C5—C4—H4	119.9	H7A—C7—H7C	109.5
C3—C4—H4	119.9	H7B—C7—H7C	109.5
			
C8—N1—C1—C6	−80.39 (18)	N1—C1—C2—C3	177.81 (13)
C7—N1—C1—C6	91.92 (17)	C2—C1—C6—C5	−0.3 (2)
C8—N1—C1—C2	102.04 (17)	N1—C1—C6—C5	−177.81 (14)
C7—N1—C1—C2	−85.65 (17)	O1—C8—C9—Cl1	14.8 (2)
C1—N1—C8—O1	173.27 (13)	N1—C8—C9—Cl1	−165.31 (11)
C7—N1—C8—O1	1.2 (2)	C1—C2—C3—C4	−0.5 (2)
C1—N1—C8—C9	−6.6 (2)	C5—C4—C3—C2	0.8 (3)
C7—N1—C8—C9	−178.63 (14)	C3—C4—C5—C6	−0.8 (3)
C6—C1—C2—C3	0.3 (2)	C1—C6—C5—C4	0.5 (3)

### Hydrogen-bond geometry (Å, °)

**Table:**

*D*—H···*A*	*D*—H	H···*A*	*D*···*A*	*D*—H···*A*
C7—H7A···O1	0.96	2.37	2.749 (2)	103.
C2—H2···O1^i^	0.93	2.58	3.4356 (19)	154.

Symmetry codes: (i) −*x*+1, −*y*+1, −*z*+1.
